# Intraperitoneal Urinary Bladder Perforation Observed in a Patient with an Indwelling Urethral Catheter

**DOI:** 10.1155/2013/765704

**Published:** 2013-09-04

**Authors:** Soichiro Ogawa, Tomonori Date, Osamu Muraki

**Affiliations:** Department of Urology, Fujita General Hospital, Kunimi-Town, Fukushima 969-1793, Japan

## Abstract

This report describes a rare case of an 86-year-old man with an indwelling urethral catheter who developed severe abdominal pain and was diagnosed with intraperitoneal urinary bladder perforation. A home-visiting nurse suspected catheter obstruction and performed a catheter exchange. However, bladder irrigation could not subsequently be performed. Computed tomography of the abdomen and pelvis after transurethral perfusion of contrast medium demonstrated extravasation of the contrast material into the peritoneal cavity. Furthermore, the Foley catheter balloon was positioned in the peritoneal cavity through the bladder. The patient was diagnosed with peritonitis due to spontaneous intraperitoneal perforation of the urinary bladder, and exploratory laparotomy was performed. During exploration, a perforated tear at the top of the bladder was discovered where the Foley catheter had penetrated the bladder. The Foley catheter balloon was floating freely in the peritoneal cavity. There was no evidence of pathologic lesions, such as cancer or inflammatory mass at the site of the injured peritoneum. Successful closure of the damaged peritoneum and bladder was performed. Since the proportion of elderly individuals continues to increase in the general Japanese population, the incidence of the chronic Foley catheterization is expected to increase. Therefore, clinicians should be aware of this potential complication.

## 1. Introduction

Bladder perforation associated with indwelling urethral catheter is rare and can be life threatening [1], and long-term use of the urethral catheter can weaken the bladder wall. This report describes a rare case of an 86-year-old man with an indwelling urethral catheter who developed severe abdominal pain and was diagnosed with intraperitoneal urinary bladder perforation.

## 2. Case Report

An 86-year-old man was admitted to our hospital with sudden onset of abdominal pain that had persisted for a few hours. He had a past history of two abdominal surgeries at 21 years and 4 years earlier for appendectomy and reduction of bowel torsion, respectively. He also had a history of cerebral infarction that occurred more than 20 years prior, two brain surgeries for subdural hematoma removal, and neurogenic bladder that had been managed for more than 4 months with a chronic indwelling urethral catheter. 

**Figure 1:**
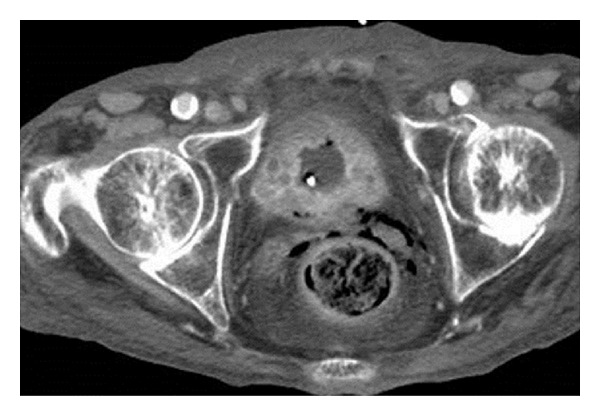
Computed tomography image showing thickening of the urinary bladder wall.

**Figure 2:**
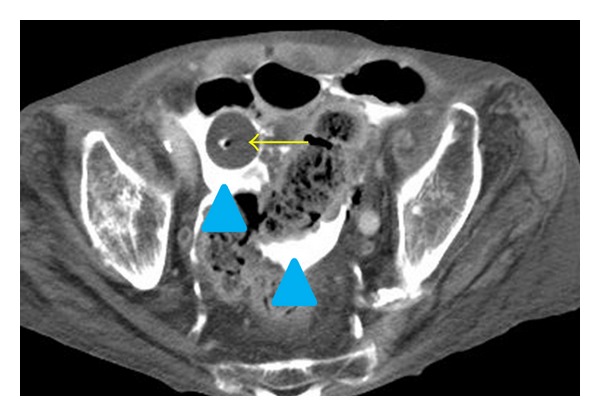
Contrast-enhanced computed tomography after transurethral perfusion of contrast medium demonstrated extravasation of the contrast material into the peritoneal cavity (▲). In addition, the Foley catheter balloon (arrowhead) was located within the peritoneal cavity through the urinary bladder.

**Figure 3:**
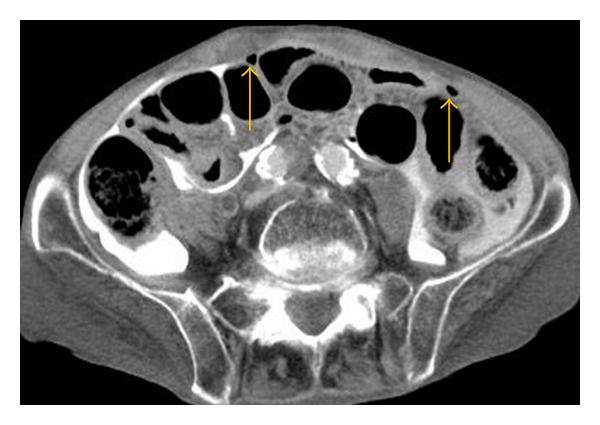
Free intraperitoneal air is identified (arrowhead).

His urethral catheter was exchanged for a new one by a home-visiting nurse. After two days, he felt acutely ill with vomiting and a decrease in urine volume. A home-visiting nurse suspected catheter obstruction and exchanged the urethral catheter for a new one. However, bladder irrigation could not subsequently be performed. At the time of his arrival to our hospital, he was conscious and alert with a pulse of 90 bpm, a blood pressure of 69/25 mm Hg, a respiratory rate of 24/min, and a temperature of 36.1°C. Physical examination showed diffuse tenderness coupled with muscle guarding and rebound pain in the lower abdomen. A 16-Fr Foley catheter had been placed. Cloudy urine was observed in his urinary drainage bag. When irrigation was attempted, there was no return. Laboratory testing revealed hemoglobin of 13.8 g/dL, hematocrit of 40.1%, leukocyte count of 7,300/mm^3^, platelet count of 517,000/mm^3^, sodium of 122 mEq/L, potassium of 4.7 mEq/L, and normal renal function. A contrast-enhanced computed tomography (CT) scan of his abdomen and pelvis after transurethral perfusion of contrast medium showed extravasation of the contrast material into the peritoneal cavity. Further, the Foley catheter balloon was placed in peritoneal cavity through the urinary bladder (Figures 1, 2, and 3). Intraperitoneal free air was also observed. Based on these CT findings, a diagnosis of peritonitis due to spontaneous intraperitoneal perforation of the urinary bladder was made. However, we could not rule out bowel perforation at that time. Therefore, we performed exploratory laparotomy. A penicillin-based antibiotic (ampicillin/sulbactam) was administered.

**Figure 4:**
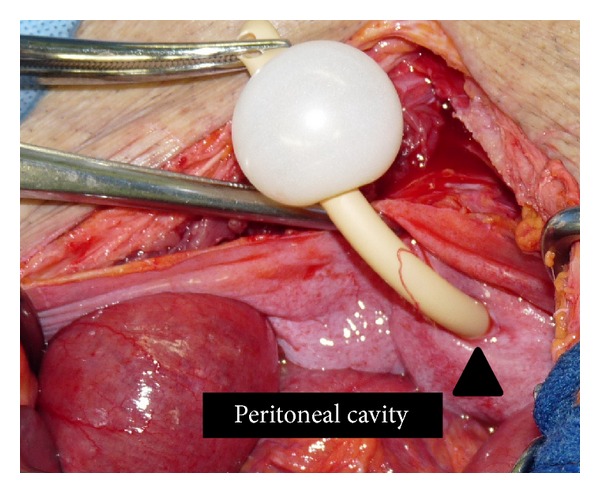
A perforated tear of about 6 mm was discovered at the dome of the urinary bladder (▲)  where the Foley catheter was penetrating. The Foley catheter balloon was floating freely in the peritoneal cavity.

During exploration, the peritoneal cavity was found to be filled with a medium amount of opacified fluid. A perforated tear of about 6 mm was discovered at the dome of the urinary bladder  where the Foley catheter was penetrating the bladder. The Foley catheter balloon was floating freely in the peritoneal cavity (Figure 4). The intestines were dilated, but no other intraperitoneal injuries were found to explain the free air. There was no evidence of pathologic lesions, such as cancer or inflammatory mass, at the site of the injured peritoneum. We concluded that the intraperitoneal air must have entered through the tear in the urinary bladder. The damaged portion of the urinary bladder was intraperitoneally repaired in a standard two-layer fashion using absorbable sutures (3-0 Polysorb, Covidien, MA, USA). The perforated peritoneum was also closed using 3-0 Polysorb in an interrupting suture technique. An extraperitoneal suprapubic cystostomy catheter was placed in addition to a urethral catheter. Successful closure was confirmed by the absence of leakage when filling the bladder to capacity. The surgical field was abundantly washed with saline solution. Drainage tubes were placed in the vesicorectal and Morison's pouch.

Postoperatively, the cystostomy drainage catheter was removed. The patient's recovery was complicated by septic shock, decubitus ulcer, limb necrosis, and pneumonia, resulting in prolonged hospitalization. A trial of discontinuation of the urethral catheter was unsuccessful. The patient was discharged 97 days after admission with an indwelling urethral catheter.

## 3. Discussion

Complications associated with the indwelling urethral catheter include bladder stones, urinary tract infection, bleeding, and iatrogenic hypospadias [2]. Among these complications, bladder perforation is rare but can be life threatening, similar to other traumatic and iatrogenic injuries [1].

The patient in the present case had a history of cerebral infarction that occurred more than 20 years earlier and was generally confined to bed because of disuse syndrome. Thus, he required long-term urethral catheterization.

Intraperitoneal perforation of the urinary bladder typically manifests with abdominal pain with guarding due to peritonitis [3]. However, a diagnosis of urinary bladder perforation is often difficult, because the symptoms are nonspecific and vague [4]. Sometimes a discrepancy between bladder irrigation and recovery of saline through the Foley catheter can be suggestive of bladder perforation [3, 5]. In the patient's case, bladder irrigation was unsuccessful, leading us to suspect bladder perforation. Of note, the patient had developed severe abdominal pain, reduction in urine output, and vomiting before a home-visiting nurse replaced the urethral catheter. Therefore, we suspect that bladder injury likely occurred before exchange of the urethral catheter. However, the Foley catheter balloon was likely advanced through the perforation with exchange of the catheter. 

Urinary bladder perforation can occur spontaneously in the setting of a weakened bladder wall. Indeed, radiation injury, bladder tumors, urinary tract infections, catheter obstruction, and long-term catheterization can damage the bladder and decrease bladder compliance [6]. Aging is also associated with increased pressure within the bladder, which can promote perforation. Bladder perforation can also result from trauma to the bladder wall [7]. However, in this case, the patient did not experience any bladder trauma. In addition, during the operation, there were no gross signs of tumors around the damaged peritoneum and bladder wall. Since there was no definite causative factor for the bladder perforation in this case, we believe that spontaneous perforation occurred.

Bladder perforation was diagnosed by CT in this case. Quagliano et al. stated that CT scan is the standard tool for evaluation of abdominal injuries [8]. However, other studies suggest that retrograde cystogram is the best method to diagnose bladder injuries [9, 10]. Appropriately selecting CT scan or cystogram is important in proper diagnosis of bladder injuries [1].

In general, intraperitoneal urinary bladder rupture requires immediate surgical treatment because it can lead to deadly peritonitis [3], which is less likely to heal with only catheter drainage [11]. Recent studies have described laparoscopic repair of intraperitoneal urinary bladder rupture. Marchand et al. stated that laparoscopic repair should be performed only in a stable patient with an isolated bladder rupture or minimal other injuries [10]. In the present case, we could not rule out perforation of the digestive tract. In addition, the patient's condition was poor. Therefore, we elected to perform open exploratory surgery.

In conclusion, this report described a rare case of spontaneous bladder perforation. Since the proportion of elderly individuals continues to increase in the general Japanese population, the incidence of the chronic Foley catheterization is expected to increase. Therefore, clinicians should be aware of this potential complication as well as other indwelling urethral catheter-related complications.
